# Acquired Elastotic Hemangioma: Case Series and Comprehensive Literature Review

**DOI:** 10.7759/cureus.1994

**Published:** 2017-12-28

**Authors:** Philip R Cohen, Brian R Hinds

**Affiliations:** 1 Department of Dermatology, University of California, San Diego

**Keywords:** acquired, angioma, elastosis, elastotic, exposed, forearm, hemangioma, solar, sun, vascular

## Abstract

Background

Acquired elastotic hemangioma is a benign vascular proliferation that typically presents as an asymptomatic red plaque on a sun-exposed site of an adult.

Material and Methods

The PubMed database was used to search the following words: acquired, angioma, arm, basal, carcinoma, cell, elastosis, elastotic, exposed, forearm, hemangioma, solar, sun, and vascular. The relevant papers and reference cited generated by the search were reviewed. The features from a case series of 11 patients with acquired elastotic hemangioma are presented. In addition, a comprehensive review of the characteristics of this unique hemangioma—not only in our 11 patients but also in the previously reported 34 individuals with this lesion—is provided.

Results

Acquired elastotic hemangioma, reported in 45 patients (24 women and 21 men), typically appeared as an asymptomatic solitary red plaque in sun-exposed areas—most commonly the forearm--of adults aged 50 years or older. The pathology shows a proliferation of vascular channels—surrounded and intertwined by intense solar elastosis--in the upper dermis, located parallel to the overlying epidermis, and separated from it by a zone of normal-appearing superficial papillary dermis. There was extensive solar elastosis surrounding and between the new blood vessels; some of the endothelial cells protrude (in a hob-nail pattern) into the vessel lumen. The clinical differential diagnosis includes basal cell carcinoma and the pathologic differential diagnosis includes other benign, malignant, and reactive vascular lesions. Ultraviolet radiation may contribute to the pathogenesis of this hemangioma since it occurs on sun-exposed sites. There was no recurrence of the lesion following either excision or observation.

Conclusions

The possibility of acquired elastotic hemangioma should be considered by clinicians when they encounter an older individual with a new red plaque on a sun-exposed site that clinically appears to be a superficial basal cell carcinoma.

## Introduction

Acquired elastotic hemangioma is a benign vascular proliferation that typically presents in older individuals on sun-exposed sites, such as the forearm. This lesion was initially described in six women in 2002 [1]; since then, it has only been reported in 28 additional patients [2-7]. A case series of 11 individuals with acquired elastotic hemangioma are described and the features of this lesion, including all 45 patients, are reviewed.

## Materials and methods

Eleven Caucasian patients (consisting of six men and five women) who were evaluated at the Department of Dermatology at the University of California San Diego from March 25, 2014 to October 11, 2017 were diagnosed with an acquired elastotic hemangioma. Clinical information was obtained from the pathology request form or the office notes or both. The following data were recorded for each patient: age, race, sex, lesion characteristics (such as size, appearance, clinical diagnosis, and duration), skin changes, lesions and/or cancers associated with sun exposure, other skin conditions, and systemic disorders.

## Results

At the time of diagnosis, the men ranged in age from 57 to 81 years (median: 73 years) and the women ranged in age from 56 to 75 years (median: 70 years). Overall, the patients ranged from 56 to 81 years (median: 72 years) (Table 1).

**Table 1:** Clinical Features of 11 Patients with Acquired Elastotic Hemangioma Abbreviations: Adeno: adenoma; AEH: acquired elastotic hemangioma; AK: actinic keratosis; AMM: amelanotic melanoma; Appear: appearance of skin lesion (color and morphology); BCC: basal cell carcinoma; BLK: benign lichenoid keratosis; BPH: benign prostatic hypertrophy; BSD: bone (and disc) spinal disease; Choleli: cholelithiasis; CD30+: CD30+ lymphoproliferative disorder; CIC: chronic interstitial cystitis; Clin Dx: clinical diagnosis submitted on pathology requisition; Def, defined; DM: diabetes mellitus; DMC: digital mucous cyst; DN: dysplastic nevus; Dys Dis: dysthymic disease; Dur: duration of lesion prior to biopsy; FDE: fixed drug eruption; Fl Top: flat topped; FP: fibrous papule; Gang: ganglion; gBCC: giant basal cell carcinoma; GERD: gatroesophageal reflux disease; HLPD: hyperlipidemia; HSV: herpes simplex virus infection; HTN: hypertension; HypoT: hypothyroidism; Irr: irritated; ISK: inflamed seborrheic keratosis; Kera: keratotic; KS: Kaposi sarcoma; LP: lichen planus; LPPD: lichenoid pigmented purpuric dermatitis; M: man; Mi: milia; MM: malignant melanoma; mon: months, mm: millimeters; Nephrolith: nephrolithiasis; Nev: nevus; NS: not stated; Osteo: osteoporosis; Pan C: pancreatic cyst; Poik: poikiloderma; PSK: purpuric seborrheic keratosis; Pulm Nod: pulmonary nodule; RAD: reactive airway disease; Ret E: Retrograde ejaculation (secondary to finasteride treatment for benign prostatic hypertrophy); SCC: squamous cell carcinoma; SCCis: squamous cell carcinoma in situ; SD: seborrheic dermatitis; SK: seborrheic keratosis; Sub Hem: subungual hematoma; Sun Expo: sun exposure-related condition, lesion, or skin cancer; TAD: transient acantholytic dermatosis; TFFD: terra firma forme dermatosis; Thy N: thyroid nodule; TP: tinea pedis; TU: tinea ungium; Var: varicose; Ver Vulg: verruca vulgaris; W: woman, W Circ: well circumscribed

C	A S	Site	Size mm	Appear	Clin Dx	Dur mon	Sun Expo	Skin Conditions	Systemic Disorders
1	56 W	Left upper arm	4 x 4	Pink papule	AK, AMM, Irr nevus	1	MM, Poik	HSV, Nev Spilus, SD, SK	Dys Dis, HypoT
2	57 M	Right forearm	8 x 8	Ill-def pink plaque	BCC	6	AK	Angioma, Nevi, SK, TP	Prostate Ca
3	63 M	Right forearm	12 x 20	Red dermal plaque	AEH	4	AK, gBCC	Nevi, Plantar verruca	HTN
4	65 W	Right calf	10 x 10	Red dermal plaque	BCC, Bite, CD30+, SCCis	4	AK, BCC	HSV, Nevi, SD, SK, TFFD	CD30+, HLPD, Osteo, RAD
5	70 W	Right forearm	8 x 8	Red plaque	NS	3	Poik, SCC	DN, HSV Mi, Nevi SK, TU, Ver Vulg	Choleli, CIC, Colon Adeno, DM, Gang, HTN, Osteo
6	72 M	Right upper arm	6 x 6	Red thin papule	BLK, ISK	4	AK, Poik	Angioma, Lentigo, SK, TAD, Xerosis	BPH, BSD, HLPD, HTN, Pan C, RAD, Ret E, Thy N
7	73 W	Left forearm	15 x 15	Purple plaque	FDE, KS, LP, LPPD	1	None	SK	DM, HTN, HLPD, HypoT
8	73 M	Left forearm	4 x 4	Red, w circ, fl top papule	Angioma, PSK	2	AK BCC MM SCC	Anal condyloma, SK, Xerosis	Colon Polyp, DM, Nephrolith
9	75 W	Right medial distal leg	6 x 6	Kera red plaque	BCC	6	AK, BCC, SCCis	Angioma, DMC, SK, TU, Var veins	Osteo
10	78 M	Left post-auricular	10 x 5	NS	BCC	NS	BCC	NS	NS
11	81 M	Right upper arm	>4 x 4	Red-purple patch	Solar purpura, vasculitis	2	AK	Angioma, Dermatitis, FP, Nevi, Sub Hem	GERD, Osteo, Pulm Nod

The asymptomatic hemangiomas were located on either the right (seven patients) or left (four patients) side of the body. The most common sites were the upper extremity (eight patients), distal lower extremity (two patients), and the postauricular area (one patient). The forearm was the most common site (five patients) followed by the upper arm (three patients).

The lesions ranged in size from 4 x 4 mm to 12 x 20 mm; the median size was 8 x 8 mm. They were red (six patients), pink (two patients), purple (one patient), or red-purple (one patient); the color was not provided for one of the individuals. The most common morphology was a plaque (six patients)—two of these patients’ lesions were described as dermal plaques. Other morphologies included papules (three patients) and a patch (one patient); the morphology was not stated for one patient.

Clinical diagnoses were provided on the pathology requisition, the office visit note, or both for 10 of the patients; a total of 21 diagnoses (of 18 different conditions) were submitted. The number of diagnoses in the differential ranged from one to four (median: two diagnoses). Basal cell carcinoma (four patients) and seborrheic keratosis (two patients with either benign lichenoid keratosis and irritated seborrheic keratosis or purpuric seborrheic keratosis) were the most common diagnoses. Other alternative diagnoses, based on the appearance of the lesion (each in one patient), included actinic keratosis, amelanotic melanoma, angioma, bite, CD30+ lymphoproliferative disorder, fixed drug eruption, irritated nevus, Kaposi sarcoma, lichen planus, lichenoid pigmented purpuric dermatosis, solar purpura, squamous cell carcinoma in situ, and vasculitis. One clinician correctly suggested the diagnosis of acquired elastotic hemangioma.

All of the acquired elastotic hemangiomas had similar pathologic features. Within the upper reticular dermis, there was an increased number of bland-appearing, thin-walled vessels arranged parallel to the overlying epidermis. Solar elastosis, usually severe, was present around the vascular channels and in the intervening dermal stroma. In addition, each only in one woman, either a perivascular lymphocytic infiltrate (Table 1, Case 1) or extravasated erythrocytes and focal hemosiderin (Table 1, Case 9) were also present.

The acquired elastotic hemangioma appeared between one to six months (median: four months) prior to evaluation; the lesion duration was not described in one patient. Ten of the patients had a history of skin changes, lesions, and/or cancer suggestive of moderate to severe sun exposure, including poikiloderma (three patients), actinic keratoses (seven patients), basal cell carcinoma (five patients, one of whom had a giant tumor, a 10 x 8 x 2.5 cm mass on his upper back), squamous cell carcinoma (three patients, one of whom had an in situ tumor), and malignant melanoma (two patients). The number of sun-related conditions per patient ranged from one to four (mean: two conditions); more commonly, there were either two conditions (five patients) or one condition (three patients) and only one patient had either three or four conditions.

The patients with acquired elastotic hemangioma also had other skin conditions and systemic disorders; whether these conditions and disorders are etiologically related to the development of acquired elastotic hemangioma remains to be determined. These included benign skin lesions (such as angioma, cysts, nevi, and seborrheic keratoses), dermatophyte infections (such as onychomycosis and tinea pedis), dermatoses (such as dermatitis, seborrheic dermatitis, terra firma forme dermatosis, transient acantholytic dermatosis, and xerosis), varicose veins, and viral infections (such as condyloma, herpes simplex virus, and verruca vulgaris). They also included benign lesions and tumors (such as benign prostatic hypertrophy, colon adenoma, ganglioma, pancreactic cyst, pulmonary nodule, and thyroid nodule), bone diseases (such as osteoporosis and spine disease), chronic interstitial cystitis, drug reactions (such as finasteride-associated retrograde ejaculation), endocrine disorders (such as diabetes mellitus and hypothyroidism), gastroesophageal reflux disease, hyperlipidemia, hypertension, neoplastic conditions (such as CD30+ lymphoproliferative disorder and prostate cancer), osteoporosis, reactive airway disease, and stone disease (cholelithiasis and nephrolithiasis).

## Discussion

History

Luis Requena (from Madrid, Spain) along with Heinz Kutzner and Thomas Mentzel (two colleagues from Friedrichshafen, Germany) provided the original description of a new variant of cutaneous hemangioma in the September 2002 issue of the Journal of the American Academy of Dermatology (Table 2) [1-7]. They described the clinical, pathological, and immunohistochemical features of a solitary vascular lesion in six women between the ages of 59 to 66 years (median age: 64 years) that was located on sun-damaged skin of the extensor forearm (five patients) or lateral neck (one patient); the endothelial cells of all of the lesions’ capillaries stained strongly positive for CD31 and CD34 (markers for blood vessel differentiation). In order to emphasize the clinicopathologic characteristics of this hemangioma, they chose the descriptive name of acquired elastotic hemangioma [1].

**Table 2:** Significance of Acquired Elastotic Hemangioma Publications Abbreviations: AEH: acquired elastotic hemangioma; Nd:YAG: neodymium-doped yttrium aluminum garnet; nm: nanometers, Pub: publication

Author Reference	Pub year	Contribution
Requena, et al. [1]	2002	They designated AEH as the descriptive name for a hemangioma variant of a new vascular proliferation. They provided a clinical, histopathologic, and immunohistochemical study of six women with a solitary vascular lesion on the sun-damaged skin of the forearm (five patients) or lateral neck (one patient).
Martorell- Calatayud, et al. [2]	2010	They presented the largest series of 14 AEH patients (eight men and six women); eight of the patients’ lesions were on the forearm. They proposed a lymphatic origin for AEH since the endothelial cells from nine of 10 lesions studied expressed a marker of lymphatic endothelial cell differentiation: D2-40 (podoplanin).
Tong and Beer [3]	2010	They presented 10 patients (six men and four women) with AEH. They suggested that AEH was derived from blood—instead of lymphatic—vascular lineage since only one of 10 lesions showed positive staining for D2-40.
Tillman, et al. [4]	2015	They authored a case report of a woman with seven new AEH her bilateral upper extremities over five years. The lesions appeared after initiating progesterone therapy; new lesions stopped developing and some lesions showed mild regression after stopping the progesterone.
Hicks and Katz [5]	2016	They reported the first description of the dermatoscopic features of AEH in a case report of a man with a lesion on the dorsum of his hand.
Mendieta- Eckert, et al. [6]	2017	They described the successful dual-wavelength vascular laser treatment of AEH on the cleavage area of a woman’s chest. The case report discussed that each of the three treatment sessions (every six weeks) consisted of using a pulsed dye (585 nm) laser followed by a Nd:YAG (1,064 nm) laser. Examination after five months of treatment--two weeks after her third treatment--showed that a complete response, without recurrence, had been achieved.
Ben Rejeb, et al. [7]	2017	Their case report described a woman with AEH on her right cheek that was clinically interpreted as a basal cell carcinoma and treated with an excisional biopsy.
Cohen and Hinds (current report)	2017	They presented a case series of 11 patients (six men and five women) with AEH on sun-exposed skin of the upper extremity (eight patients), lower extremity (two patients), or postauricular area (one patient) and provided a comprehensive review of this benign vascular lesion. Their series increased the number of reported AEH patients by 32% (11/34); the number of individual described also comprises 24% (11/45) of the published AEH patients. They emphasized that ultraviolet radiation may have an etiologic role in the pathogenesis of AEH and show that 91% (10 of 11) of their patients had a history of skin changes, lesions, and/or cancer suggestive of moderate to severe sun exposure.

It was not until eight years later that Martorell-Calatayud, et al. reported—in the April 2010 issue of the Journal of Cutaneous Pathology—the features of acquired elastotic hemangioma in a series of 14 patients: eight men and six women, ranging in age from 51 to 82 years (median age: 65 years). The solitary lesions were located on the forearm (eight patients), lower lip (two patients), hand (one patient), nostril (one patient), and shoulder (one patient); the site was not specified in one patient. They proposed that this acquired vascular proliferation was of lymphatic origin based on the expression of D2-40 (podoplanin, a specific marker of lymphatic differentiation) in nine of the 10 lesions they studied [2].

That same year—in the December 2010 issue of the Journal of Cutaneous Pathology—Tong and Beer reported 10 additional patients (six men and four women) with a solitary acquired elastotic hemangioma on either the arm (nine patients) or upper chest (one patient). The patients were in good health, had no history of immunosuppression, and ranged in age from 50 to 79 years (median age: 59.7 years). The authors demonstrated that the lymphatic endothelial marker D2-40 was positive in only one of the 10 lesions, which suggested that most of the acquired elastotic hemangiomas were of a blood vessel—and not lymphatic vascular—lineage [3].

Half a decade would pass before the next report of an acquired elastotic hemangioma. Tillman, et al. had a resident poster presentation at the fall meeting of the American Osteopathic College of Dermatology that was held in Orlando, Florida from October 15-18, 2015. They presented a 57-year-old woman who developed multiple lesions on both of her upper extremities after initiating progesterone therapy. The report was subsequently published in the December 2015 issue of Practical Dermatology [4].

In the following year, a case report by Hicks and Katz, highlighting the dermatoscopic features of an acquired elastotic hemangioma on the dorsal hand of a man in his 60’s, was published in the April 2016 issue of Dermatology Practical and Conceptual [5]. Subsequently, two additional patients with this vascular lesion have been reported in 2017 [6-7]. In March 2017, the successful vascular laser treatment of an acquired elastotic hemangioma located on the cleavage of a 65-year-old woman by Mendieta-Eckert, et al. was published in Dermatology Surgery [6]. Also, in the October 2017 issue of Our Dermatology Online Journal, Ben Rejeb, et al. described a 64-year-old woman with an acquired elastotic hemangioma on her right cheek [7].

We currently present a series of 11 additional patients (six men and five women) with a solitary acquired elastotic hemangioma. They range in age from 56 to 81 years (median age: 72 years). The vascular lesions were located on their upper extremity (eight patients), lower extremity (two patients), and postauricular (one patient).

Epidemiology

Acquired elastotic hemangiomas have been described in 45 patients: 24 women and 21 men [1-7], as well as the current report (Tables 3-4); hence, the lesion occurs slightly more often in women than men with the women to men ratio of eight to seven. Their age—when the diagnosis was established--ranged from 50 to 82 years (median: 63 years). At the time of diagnosis, the women ranged in age from 50 to 76 years (median: 61 years) and the men ranged in age from 53 to 82 years (median: 68 years).

**Table 3:** Clinical Characteristics of Women with Acquired Elastotic Hemangioma Abbreviations: AMM: amelanotic malignant melanoma; BCC: basal cell carcinoma; C: case; CD30+ LD: CD 30+ lymphoproliferative disorder; CR: current report; FDE: fixed drug eruption; L: left; LH: lymphoid hyperplasia; LP: lichen planus; LPPD: lichenoid pigmented purpuric dermatosis; PAT: purpura annularis telangiectoides; R: right; Ref: reference; SCCis: squamous cell carcinoma in situ; THH: targetoid hemosiderotic hemangioma ^1^The woman had a total of seven erythematous, well-defined, non-tender, slightly elevated, non-blanching plaques on her arms bilaterally; the largest was on the right lower forearm. ^2^The single lesion was localized in her cleavage.

C	Age (yr)	Location	Clinical diagnosis	Ref
1	50	R forearm	Actinic keratosis, squamous cell carcinoma	[3] C7
2	51	L forearm	BCC	[2] C4
3	53	Not stated	Not stated	[2] C10
4	55	L forearm	BCC (superficial)	[2] C5
5	55	L forearm	BCC	[2] C11
6	55	R upper arm	Not stated	[3] C2
7	56	L upper arm	AMM, angioma, inflamed nevus	CR1
8	57	R forearm	BCC	[2] C3
9	57	R and L forearm and arm^1^	Kaposi sarcoma, PAT, THH	[4]
10	59	R forearm	SCCis	[1] C3
11	60	L forearm	BCC	[1] C5
12	60	L clavicle	BCC	[3] C3
13	61	L biceps	Vascular lesion	[3] C8
14	63	L forearm	Lupoid lesion	[1] C1
15	64	R neck (lateral)	BCC	[1] C6
16	64	R cheek	BCC	[7]
17	65	Chest (central)^2^	Hemangioma, inflammatory lesion, LH	[6]
18	65	R calf	BCC, bite, CD30+ LD, SCCis	CR4
19	66	L forearm	Erythema, hemangioma	[1] C2
20	70	R upper arm	Vascular lesion	[2] C12
21	70	R forearm	Not stated	CR5
22	73	L forearm	FDE, Kaposi sarcoma, LP, LPPD	CR7
23	75	R distal leg (medial)	BCC	CR9
24	76	L forearm	BCC	[1] C4

**Table 4:** Clinical Characteristics of Men with Acquired Elastotic Hemangioma Abbreviations: BCC: basal cell carcinoma; BLK: benign lichenoid keratosis; C: case; CR: current report; L: left; R: right; Ref: reference

C	Age (yrs)	Location	Clinical diagnosis	Ref
1	53	L forearm	BCC, squamous cell carcinoma in situ	[3] C4
2	54	R forearm	BCC	[3] C1
3	57	R forearm	BCC	CR2
4	59	L forearm	BCC, lichenoid keratosis, nevus	[3] C10
5	60’s	L hand (dorsal)	Granuloma annulare	[5]
6	61	R upper arm	Not stated	[3] C6
7	63	Lower lip	BCC	[2] C6
8	63	R forearm	Acquired elastotic hemangioma	CR3
9	65	R hand (dorsum)	BCC	[2] C14
10	65	L forearm	Not stated	[3] C5
11	68	R nostril	BCC	[2] C9
12	69	L forearm	BCC	[2] C1
13	70	R forearm	BCC	[2] C13
14	71	R forearm	Ecchymosis	[2] C2
15	72	R upper arm	BLK, irritated seborrheic keratosis	CR6
16	73	L forearm	Angioma, purpuric seborrheic keratosis	CR8
17	76	L lip	Vascular lesion	[2] C8
18	78	L postauricular	BCC	CR10
19	79	L shoulder	BCC (with dilated blood vessels)	[3] C9
20	81	R upper arm	Solar purpura, vasculitis	CR11
21	82	L shoulder	BCC	[2] C7

The lesion is usually asymptomatic. However, at least two patients noted the lesion to be painful: one of the women described by Requena, et al. [1] and a 57-year-old woman right forearm acquired elastotic hemangioma (Table 3, Case 8) [2]. None of the patients had a history of trauma, previous procedure, or radiotherapy at the site of their vascular lesion.

Clinical presentation

**Figure 1:**
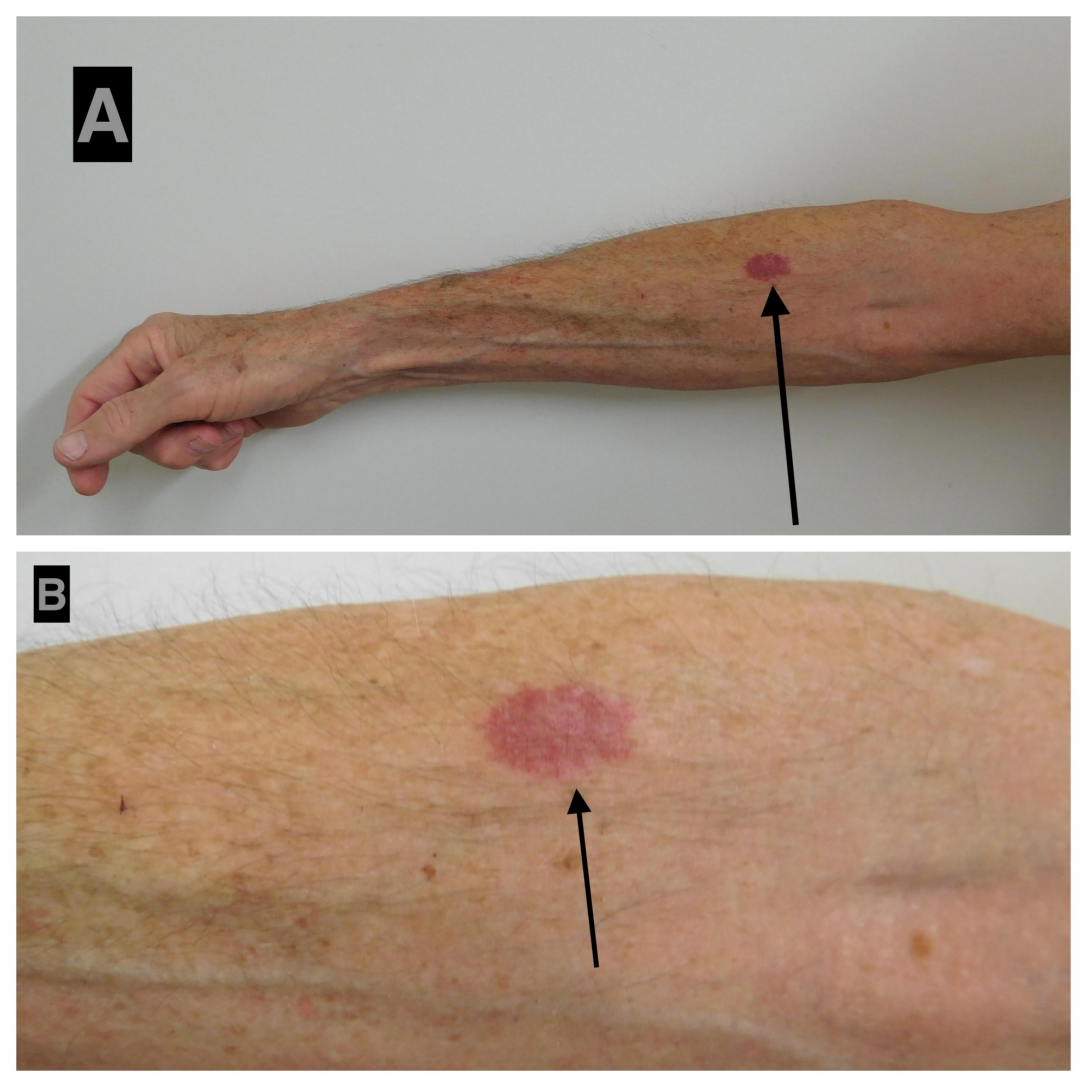
Acquired elastotic hemangioma: right forearm of a 63-year-old man Distant (A) and closer (B) views of an asymptomatic 12 x 20 mm red dermal plaque on his right forearm of four months duration. The man had a history of hypertension, actinic keratoses (that had been treated with liquid nitrogen cryotherapy), and a giant (10 x 8 x 2.5 cm) basal cell carcinoma on his upper central back (that had been successfully treated with a combination of vismodegib and nivolumab). According to the patient, the lesion began as a ‘pimple’ that he picked; the affected area enlarged and remained as a red lesion beneath his skin. The clinical differential diagnosis of the right forearm lesion, acquired elastotic hemagioma, was confirmed by the pathology features observed on the biopsy of the lesion: variable-sized compact vascular channels lined by cytologically bland endothelial cells in the superficial dermis; elastosis was present around the vascular channels and in the intervening dermal stroma.

Acquired elastotic hemangiomas typically present as solitary pink to red to purple, ill-defined yet well-demarcated, slowly growing patches or plaques (Figure 1). However, one woman had a total of seven acquired elastotic hemangiomas on her upper extremities [4]. The individual lesions range in size from 4 x 4 mm (Table 1, Case 1) to 5 x 5 cm [1].

Acquired elastotic hemangiomas are diascopy-negative. Diascopy refers to using a clear material, such as a glass microscope slide, to press against a skin lesion to assess for blanchability; some vascular lesions, such as telangiectasias, acquire a blanched appearance after the lesion has been depressed since the blood dissipates intravascularly [8]. Blanchability of acquired elastotic hemangiomas was evaluated in 21 patients; none of the lesions blanched under diascope pressure [1-2, 7].

Acquired elastotic hemangioma frequently occurs on sun-exposed areas. They occurred on the left side (22 patients: 12 women and 10 men) and on the right side (21 patients: 11 women and 10 men) of the body; one woman, with multiple hemangiomas, had lesions on both sides of her body [4]. Lesions also occurred on the lower lip (one man) and the central chest cleavage area (one woman). 

**Figure 2:**
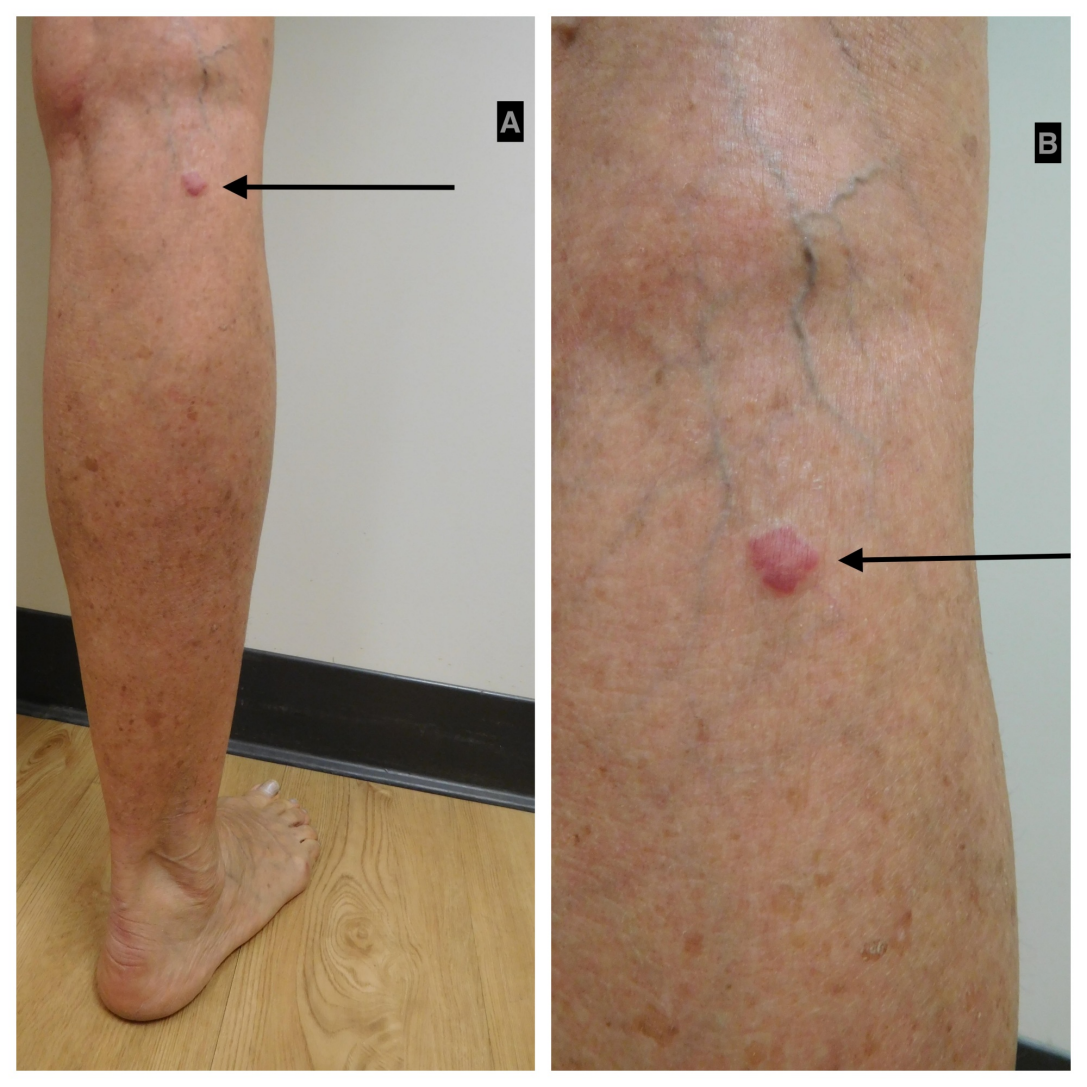
Acquired elastotic hemangioma: right calf of a 65-year-old woman Distant (A) and closer (B) views of an asymptomatic 10 x 10 mm red dermal plaque on the superior aspect of the right calf of four months duration. The woman had a history of a CD30-positive lymphoproliferative disease involving her left leg that was diagnosed 17 years earlier and successfully treated with radiotherapy nine years ago without recurrence. She also had a history of hyperlipidemia, osteoporosis, and reactive airway disease. In addition, she previously had several basal cell carcinomas that had been excised and actinic keratoses that had been treated with liquid nitrogen cryotherapy. She was unaware of the right calf lesion; however, it had not been present at her total body skin check four months earlier. The clinical differential diagnosis of the right calf lesion included arthropod bite reaction, basal cell carcinoma, CD30-positive lymphoproliferative disorder, and squamous cell carcinoma in situ. The diagnosis of acquired elastotic hemangioma was established by the changed note on the lesion biopsy: multiple small capillaries situated in the superficial dermis amidst elastotic material.

**Figure 3:**
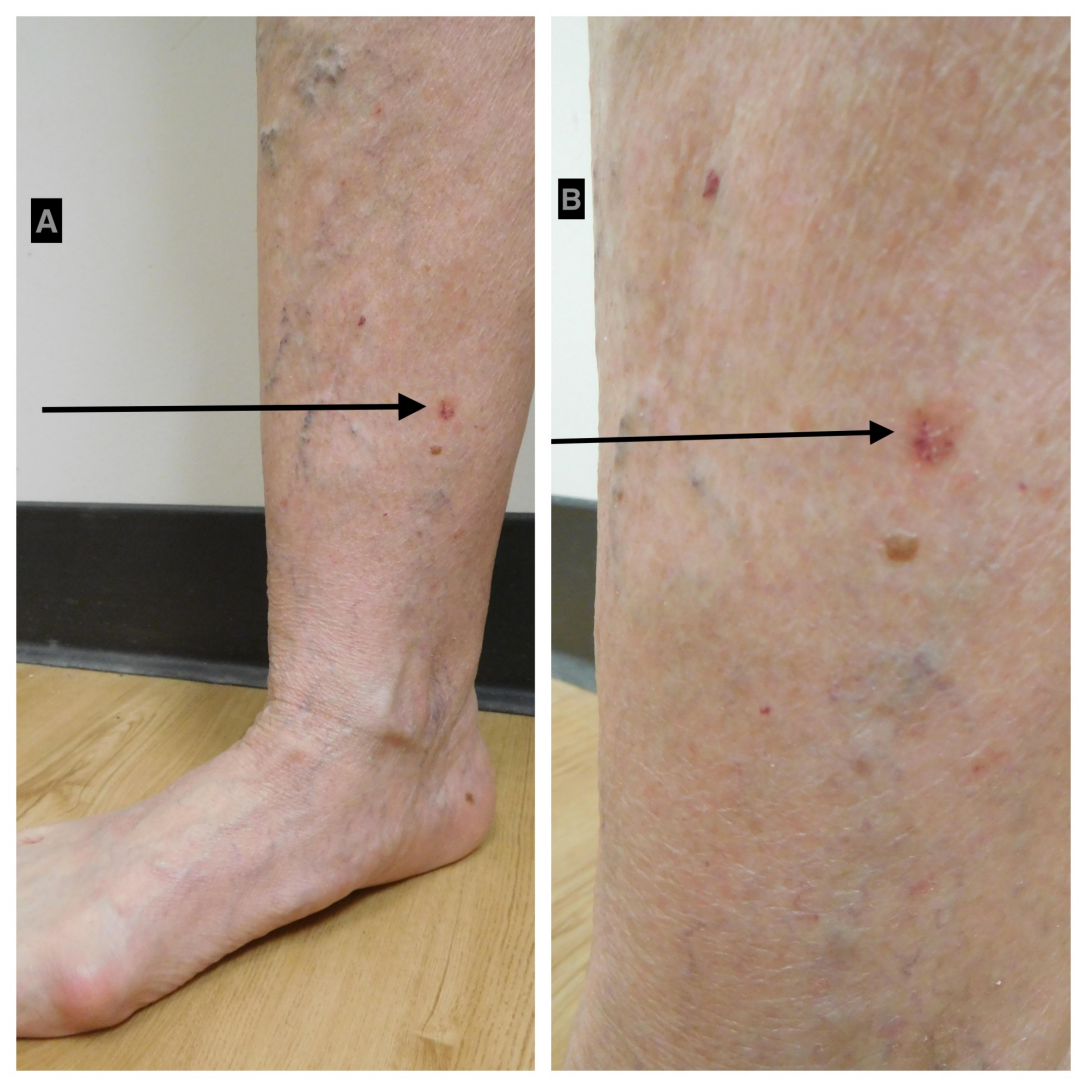
Acquired elastotic hemangioma: right medial distal leg of a 75-year-old woman Distant (A) and closer (B) views of an asymptomatic 6 x 6 mm keratotic red plaque on the right medial distal leg of six months duration. The woman had a history osteoporosis. She also had a history of basal cell carcinoma and squamous cell carcinoma that were excised and actinic keratoses that had been treated with liquid nitrogen cryotherapy. The clinical differential diagnosis of the right medial distal leg lesion was basal cell carcinoma. An excision shave biopsy was performed; the diagnosis of acquired elastotic hemangioma was established by the pathology findings: an increase in vascular lobules within the dermis with extensive solar elastosis; focal hemosiderin and extravasated red blood cells were also present.

The most common location of an acquired elastotic hemangioma was an upper extremity (32 patients, 71%) (Table 5) [1-7] and the current report. The forearm was more frequently involved (23 patients) than the upper arm (seven patients), the dorsal hand (two patients), and the biceps (one patient); one woman had lesions on both her forearm and upper arm (Table 3, Case 9) [4]. The lesions also occurred on the head and neck (six patients), the back and chest (four patients), and the lower extremity (two patients) (Figures 2-3). The lesion location was not provided for a 53-year-old woman (Table 1, Case 3) [2].

**Table 5:** Location of Acquired Elastotic Hemangiomas in 45 Patients ^1^There are 46 locations for the 45 individuals with acquired elastotic hemangioma since the woman with seven lesions had hemangiomas located bilaterally on both the forearm and the upper arm (Table 3, Case 9) [4]. The lesion is recorded at both sites in the column for women and the column for total in the rows for forearm and upper arm. However, since the forearm and upper arm are both part of the upper extremity, the lesion is recorded only once in the column for women and the column for total in the row for total in the upper extremity section of the table.

Site	Women	Men	Total
Upper extremity			
Forearm^1^	13	10	23
Upper arm^1^	4	3	7
Hand	0	2	2
Biceps	1	0	1
Total^1^	17	15	32
Head and neck			
Lip	0	2	2
Cheek	1	0	1
Neck	1	0	1
Nostril	0	1	1
Postauricular	0	1	1
Total	2	4	6
Back and chest			
Shoulder	0	2	2
Clavicle	1	0	1
Cleavage	1	0	1
Total	2	2	4
Lower extremity (calf or distal leg)			
Total	2	0	2
Not stated			
Total	1	0	1
TOTAL	24	21	45

The location of the acquired elastotic hemangiomas was similar in women and in men. In women, acquired elastotic hemangiomas most commonly appeared on the forearm (13 patients), the upper arm (four patients), and the leg (two patients); as previously mentioned, one of the women had lesions on both her forearm and upper arm (Table 3, Case 9) [4]. Other sites, each in one woman, included the biceps, the cheek, the chest (cleavage area), the clavicle, and the neck. In men, the lesions most frequently appeared on the forearm (10 patients), the upper arm (three patients), and--in two men each—on the hand (dorsum), the lip, and the shoulder. Other sites, each in one man, included the nostril and the postauricular area.

Dermoscopy

The dermatoscopic features of acquired elastotic hemangioma were described by Hicks and Katz [5]. They evaluated an 8 x 6 mm violaceous plaque-like lesion (with a slightly raised thin border and a mildly rough surface without scale) on the left lateral dorsal hand of a man in his 60’s. The new lesion was asymptomatic and had only been noticed two weeks prior.

Non-polarizing dermatoscopy of the lesion—at standard magnification--showed a homogenous, non-pigmented violaceous plaque, without obvious vasculature; there were no keratin features or ulceration. However, polarized dermatoscopy shows prominent and widespread shiny white structures distributed evenly throughout the lesion. The investigators suggested that the shiny white areas that they observed were a specific polarization artifact that may have been caused by the horizontal band-like proliferation of capillaries with intervening collagen bundles in the superficial dermis [5].

Pathology

**Figure 4:**
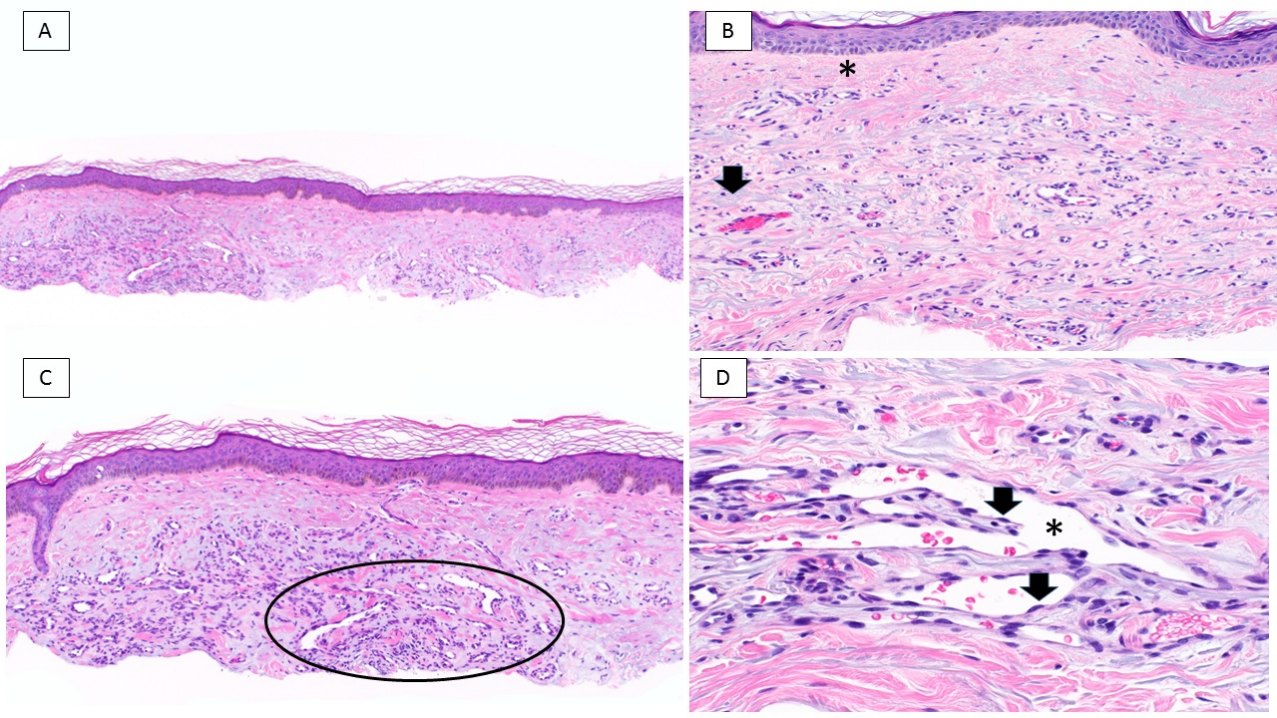
Acquired elastotic hemangioma: pathology features A low magnification view (A) of a thin shave biopsy shows sun-damaged skin of the forearm; there is a well-delineated, horizontally oriented proliferation of vascular channels. Higher magnification views (B, C, and D) highlight specific pathologic changes. There is a grenz zone showing a narrow band of the papillary dermis uninvolved by underlying pathology (asterisk); also, blue elastotic fibers (arrow) are intertwined with compactly arranged, thin-walled vascular channels (B). Some of the lesion’s blood vessels (circle) display jagged, slit-like spaces (C). Focal protrusion of endothelial cells (arrows) toward the center of the lumen of the vascular space (asterisk) is noted; the constituent endothelial cells lack nuclear atypia (D) (Hematoxylin and eosin: A, x 4; B, x 20; C, x 10; D, x 40).

Biopsy and excision specimens of acquired elastotic hemangiomas from the patients in this clinical series and the previously reported individuals showed similar pathologic changes (this series report; [1-7]); indeed, there were some papers that demonstrated the microscopic features of acquired elastotic hemangioma but did not discuss any of the clinical aspects from the photomicrographs they included [9-11]. There was a band-like proliferation of capillary blood vessels, arranged parallel to the epidermis, that was present in the superficial dermis. The endothelial cells lining the vessels were bland; they did not have cellular or nuclear atypia and there were few to no mitoses. However, in some areas, the endothelial cells showed a ‘hobnail pattern’ by protruding into the lumen of the vessels (Figure 4) [1-3].

Another characteristic feature of an acquired elastotic hemangioma was the prominent solar elastosis of the collagen bundles surrounding and intermingled with the proliferation of capillaries. In addition, the overlying epidermis was either normal or atrophic and was separated from the vascular proliferation by a grenz zone - a narrow band of the papillary dermis uninvolved by underlying pathology [12]. The presence of either a perivascular lymphocytic infiltrate (Table 1, Case 1) or extravasated erythrocytes and focal hemosiderin (Table 1, Case 9) were considered to be coincidental findings in two of the women from our case series.

Immunoperoxidase studies show uniformly showed positive staining of the endothelial cells with CD31 or CD34 or both, which are markers that support a capillary vascular lineage for acquired elastotic hemangioma [1-3, 7]. Although Martorell-Calatayud, et al. demonstrated positive D2-40 (podoplanin, a specific marker of lymphatic differentiation) in nine of the 10 lesions they studied, which was suggestive of a lymphatic origin for this vascular proliferation, a follow-up study by Tong and Beer only demonstrated positive D2-40 staining vessels in only one of the 10 cases they evaluated and CD34-positive staining vessels in all of these cases. The latter investigators concluded that most acquired elastotic hemangiomas were of blood vascular—and not lymphatic vascular—lineage [3].

In some of the acquired elastotic hemangioma lesions, the pericytes surrounding the vascular channels stained positive for alpha-smooth muscle actin [1-2, 7]. Requena, et al. observed positive staining in all of the six lesions they evaluated [1]. Similarly, Ben Rejeb, et al. noted positive staining in the lesion from their patient (Table 3, Case 16) [7]. However, Martorell-Calatayud, et al. only demonstrated positive staining in one of the 10 lesions they studied [2].

Proliferating markers (anti-mitotic proteins antibody (MPM-2) and/or Ki-67) were evaluated by two groups of researchers. Only a few nuclei of the endothelial cells of the acquired elastotic hemangioma blood vessels stained positive with MPM-2 and Ki-67 in the six lesions reported by Requena, et al. [1]. None of the 10 lesions studied by Martorell-Calatayud, et al. showed positive staining for Ki-67 [2]. The endothelial cells of the vessels were also negative for human herpes virus-8 (HHV-8) staining in one patient’s lesions (Table 3, Case 16) [7].

Differential diagnosis

The clinical differential diagnosis of acquired elastotic hemangioma includes neoplastic and benign tumors, other vascular lesions, dermatoses, and other conditions (Table 6) (current series; [1-7]). Sixty diagnoses were suggested by the clinicians who biopsied 40 of the 45 patients; there were five patients (three women and two men) for whom a clinical diagnosis was not stated. The number of diagnoses submitted ranged from one (27 patients) to four (two patients); the median number of submitted diagnoses was one. Two diagnoses were considered in seven patients and three diagnoses were entertained in four patients.

**Table 6:** Clinical Differential Diagnosis of Acquired Elastotic Hemangioma ^1^The pathology requisition slip submitted by the clinician listed one or more possible diagnoses for 40 of the acquired elastotic hemangioma patients; a clinical diagnosis was not provided for five of the patients: three women and two men. ^2^Keratoses include one of each of the following lesions: benign lichenoid keratosis, irritated seborrheic keratosis, lichenoid keratosis (not otherwise specified), and purpuric seborrheic keratosis.

Diagnosis^1^	Women	Men	Total
Neoplastic tumors or pre-neoplastic lesions			
Basal cell carcinoma	11	12	23
Squamous cell carcinoma (invasive or in situ)	3	1	4
Kaposi sarcoma	2	0	2
Actinic keratosis	1	0	1
CD30+ lymphoproliferative disorder	1	0	1
Malignant melanoma (amelanotic)	1	0	1
Total	19	13	32
Vascular lesions (benign)			
Angioma (hemangioma)	3	1	4
Vascular lesion (not otherwise specified)	2	1	3
Ecchymosis and purpura (solar)	0	2	2
Acquired elastotic hemangioma	0	1	1
Erythema	0	1	1
Targetoid hemosiderotic hemangioma	1	0	1
Vasculitis	0	1	1
Total	6	7	13
Dermatoses and other conditions			
Bite	1	0	1
Fixed drug eruption	1	0	1
Granuloma annulare	0	1	1
Inflammatory lesion (not otherwise specified)	1	0	1
Lichen planus	1	0	1
Lichenoid pigmented purpuric dermatosis	1	0	1
Lymphoid hyperplasia	1	0	1
Lupoid lesion	1	0	1
Purpura annularis telangiectoides	1	0	1
Total	8	1	9
Benign tumors (non-vascular)			
Keratoses^2^	0	4	4
Nevus (irritated and not otherwise specified)	1	1	2
Total	1	5	6
TOTAL	34	26	60

Basal cell carcinoma was the most common diagnosis (23 patients); this represented 39% (23 of 59 submitted diagnoses) of the diagnoses suggested on the pathology requisition. The next most frequent diagnoses—each listed four times—were squamous cell carcinoma (invasive or in situ), angioma (hemangioma), and benign keratosis (in three men). A vascular lesion (not otherwise specified) in three patients, and either Kaposi sarcoma or ecchymosis and purpura (solar) each in two patients were the other diagnoses suggested more than once.

The pathologic differential diagnosis of acquired elastotic hemangioma includes benign (such as acquired tufted hemangioma, angioma, and targetoid hemosiderotic hemangioma) and malignant (such as angiosarcoma, Dabska tumor, Kaposi sarcoma, and retiform hemangioendothelioma) vascular lesions (Table 7) [1-2, 4, 7, 9, 11, 13-15]. The endothelial cells that line the vascular channels in acquired elastotic hemangioma can be plump and protrude into the vessel lumina in a hobnail pattern; therefore, other vascular proliferations with a hobnail pattern (such as angiosarcoma, Dabska tumor, retiform hemangioendothelioma, and targetoid hemosiderotic hemangioma) need to be considered and excluded. Also, other vascular lesions with similar pathologic features (such as Dabska tumor and tufted hemangioma) can be excluded since they typically appear in childhood--in contrast to acquired elastotic hemangioma that presents in older individuals.

**Table 7:** Pathologic Differential Diagnosis of Acquired Elastotic Hemangioma ^1^Acquired elastotic hemangioma-like changes and eccrine sweat duct squamous metaplasia in lichen simplex chronicus and prurigo nodularis-like lesions of the knees and elbow seem to represent non-neoplastic epithelial, vascular, and eccrine sweat duct reactive changes—likely associated with chronic pressure or repeated mechanical stimulation—which have a marked predilection for the knee and elbow.

Condition	References
Acquired tufted hemangioma (angioblastoma of Nakagawa)	[1-2, 4, 7]
Acroangiodermatitis of Mali (pseudo-Kaposi sarcoma)	[1-2, 7, 9]
Angioma (cherry or senile)	[1-2, 7, 11]
Angiosarcoma (low-grade, well-differentiated)	[2, 11]
Cutaneous collagenous vasculopathy	[13-14]
Dabska tumor (endovascular papillary angioendothelioma)	[2]
Kaposi sarcoma (early patch stage)	[1-2, 4, 7, 9]
Reactive epithelial, vascular and eccrine sweat duct changes^1^	[15]
Retiform hemangioendothelioma	[2]
Stewart-Bluefarb syndrome (pseudo-Kaposi sarcoma)	[1]
Targetoid hemosiderotic hemangioma (hobnail hemangioma)	[1, 4]

In addition, the pathologic differential diagnosis of acquired elastotic hemangioma includes cutaneous collagenous vasculopathy. Clinically, cutaneous collagenous vasculopathy usually presents as asymptomatic blanchable telangiectasias on the trunk and extremities; it is morphologically similar in appearance to generalized essential telangiectasias or pigmented purpuric dermatosis. Microscopically, similar to acquired elastotic hemangioma, there are dilated blood vessels in the superficial dermis. However, in contrast to acquired elastotic hemangioma, the walls of the vessels in cutaneous collagenous vasculopathy are thickened with hyaline material that is not only periodic acid-Schiff stain-positive but also shows Type IV collagen immunoreactivity and severe solar elastosis around and between the vessels is not a characteristic feature [13-14].

Also, reactive vascular lesions—such as pseudo-Kaposi sarcoma (acroangiodermatitis of Mali and Stewart-Bluefarb syndrome)—are in the pathologic differential diagnosis. Recently, Kacerovska, et al. [15] described a reactive lesion of not only vascular elements but also epithelial and eccrine sweat ducts that occurs on the knee and the elbow—sites associated with chronic pressure or repeated mechanical stimulation. The vascular changes in these lesions are similar to those observed in acquired elastotic hemangioma; however, there is also squamous metaplasia of the eccrine ducts and epithelial changes consistent with lichen simplex chronicus and prurigo nodularis.

Pathogenesis

There is evidence that long-term sun exposure contributes to the development of acquired elastotic hemangioma [1-3, 7]. First, there was severe elastosis in the dermis of the lesions. Second, the lesions occurred on locations that were exposed to the sun—most commonly the upper extremity and, specifically, the forearm. Third, as demonstrated by 91% (10 of 11) of the patients in the current case series, many of the affected individuals had a history of skin changes, lesions, and/or cancer suggestive of moderate to severe sun exposure, including poikiloderma, actinic keratoses, basal cell carcinoma, squamous cell carcinoma, and malignant melanoma; two of the patients reported by Tong and Beer had multiple prior basal cell carcinomas [3].

Tillman, et al. raised the possibility of hormonal influence in the pathogenesis of acquired elastotic hemangioma. Their patient, a 57-year-old woman, developed seven of these lesions on both of her upper extremities during the five-year period after she began progesterone therapy. Indeed, after stopping progesterone, she not only stopped developing new acquired elastotic hemangiomas but also experienced that some of the lesions showed mild regression [4].

The patients in the current case series had several other skin conditions and systemic disorders (Table 1). Similarly, the woman with seven acquired elastotic hemangiomas also had a history of rosacea and hypertension [4]. However, an association between the pathogenesis of acquired elastotic hemangioma and any of other the skin conditions or systemic disorders that these individuals have remains to be established.

Treatment

Acquired elastotic hemangioma is a benign vascular lesion. However, prior to establishing the prognosis for this lesion, most of the tumors were excised (25 patients) [1-3, 5, 7]. However, in more recent reports (including the patients in this case series), the acquired elastotic hemangioma were biopsied to establish the diagnosis and the residual lesion was observed (19 patients) [3, 4]; one woman, with seven lesions who was monitored clinically, developed no new tumors and had mild regression of her current hemangiomas after discontinuing progesterone [4]. The remaining patient’s acquired elastotic hemangioma, which was located on the cleavage area of her chest, was successfully removed after three treatment sessions (spaced at six-week intervals) using a dual-wavelength laser system (Table 3, Case 17); she received a maximum of 9 Joules per square centimeter (spot 7 millimeters, pulse duration of 0.5 milliseconds) with a pulsed dye laser (585 nanometers) and 145 Joules per square centimeter (spot 5 millimeters, pulse duration 15 milliseconds) with a neodymium-doped yttrium aluminum garnet (Nd:YAG) (1,064 nanometers) [6].

Prognosis

All of the patients in our case series only had biopsies of their lesions without additional treatment or lesion-specific follow-up. However, follow-up was provided for 18 patients with acquired elastotic hemangioma in the literature. Whether treated with excision (10 patients) [1, 3, 5], laser (one patient) [6], or observation (seven patients) [3], none of the patients had a recurrence of their lesions. 

## Conclusions

Acquired elastotic hemangioma, a benign—usually asymptomatic--vascular proliferation, was originally described in 2002. Including the patients in this case series, it has been reported in 45 patients: 24 women and 21 men. The lesion typically appeared in sun-exposed sites of adults; the youngest individual was 50 years old and the oldest patient was 82 years old; the median onset age is 63 years. The hemangioma presented as a solitary pink to red to purple patch or plaque; however, one woman developed seven lesions after beginning progesterone therapy. The most common location is the forearm (23 patients); other frequent sites included the upper arm (seven patients), the head and neck (six patients), and the back and chest (four patients); one woman had lesions on both her forearm and upper arm. All of the lesions had similar pathology features: a proliferation of vascular channels in the upper dermis, located parallel to the overlying epidermis, and separated from it by a grenz zone in the superficial papillary dermis. There was extensive solar elastosis surrounding and between the new blood vessels, some of which showed a hobnail-like protrusion of the endothelial cells into the vessel lumen. Basal cell carcinoma was the most common clinical differential diagnosis (23 patients); the pathologic differential diagnosis included not only benign and malignant vascular tumors but also reactive vascular lesions. The predominance of lesions in areas that have been exposed to sun suggests that ultraviolet radiation has a role in the pathogenesis of acquired elastotic hemangioma. Earlier investigators did not observe recurrence of the lesion following either excision or observation. One woman’s lesion was successfully treated with dual wavelength laser therapy and another woman had mild regression of her hemangiomas (and no new lesions) after discontinuing progesterone.

In summary, we suspect that this vascular lesion occurs more frequently than the small number of publications and reported patients would indicate. Also, we recommend that clinicians entertain the possibility of this acquired hemangioma when they encounter an older individual with a new red plaque on a sun-exposed site that clinically appears to be a superficial basal cell carcinoma. The distinctive pathology observed on a biopsy of the lesion—severe solar elastosis around and within a proliferation of vessels in the upper dermis that is distributed parallel to the overlying epidermis--will establish the diagnosis.
